# HIV Community-Level Stigmatizing Attitudes in Tanzania: Perspectives from Antenatal Care

**DOI:** 10.24248/eahrj.v4i2.635

**Published:** 2020-11-26

**Authors:** Saumya S Sao, Brandon A Knettela, Godfrey A Kisigo, Elizabeth T Knippler, Haika Osaki, Rimel N Mwamba, Jane Rogathi, James S Ngochob, Blandina T Mmbaga, Melissa H Watt

**Affiliations:** a Duke Global Health Institute, Duke University, Durham, NC, USA; b Kilimanjaro Clinical Research Institute, Moshi, Tanzania; c GillingsSchool of Global Public Health, University of North Carolina, Chapel Hill, NC, USA; d Kilimanjaro Christian Medical Centre, Moshi, Tanzania; e Kilimanjaro Christian Medical University College, Moshi Tanzania; f Department of Population Health Sciences, University of Utah, Salt Lake City, UT, USA

## Abstract

**Introduction:**

Stigma significantly impacts retention in HIV care and quality of life among people living with HIV. This study explored community-level HIV stigma from the perspective of patients and healthcare workers in antenatal care (ANC) in Moshi, Tanzania.

**Methods:**

We conducted in-depth interviews with 32 women (20 living with HIV), key-informant interviews with 7 ANC clinic employees, and two focus group discussions with 13 community health workers.

**Results:**

Themes emerged related to drivers and manifestations of stigma, resilience to stigmatizing attitudes, and opportunities to address stigma in ANC. Drivers of stigma included a fear of infection through social contact and associations of HIV with physical weakness (e.g., death, sickness) and immoral behaviour (e.g., sexual promiscuity). Manifestations included gossip, physical and social isolation, and changes in intimate relationships. At the same time, participants identified people who were resilient to stigmatizing attitudes, most notably individuals who worked in healthcare, family members with relevant life experiences, and some supportive male partners.

**Conclusion/Recommendations:**

Supportive family members, partners, and healthcare workers can serve as role models for stigma-resilient behaviour through communication platforms and peer programs in ANC. Manifestations of HIV stigma show clear links to constructs of sexuality, gender, and masculinity, which may be particularly impactful during pregnancy care. The persistence of stigma emphasizes the need for innovation in addressing stigmatizing attitudes in the community. Campaigns and policies should go beyond dispelling myths about HIV transmission and immorality to innovate peer-led and couples-based stigma reduction programming in the ANC space.

## BACKGROUND

Prevention of mother-to-child transmission (PMTCT) of HIV programs, which include HIV testing and counselling for all pregnant women at their first antenatal care (ANC) visit, are an important component of comprehensive HIV care. In 2012, the World Health Organization (WHO) recommended the global adoption of the Option B+ protocol, which indicates that all pregnant women living with HIV (WLHIV) should initiate antiretroviral (ARV) therapy at the start of ANC and continue for life.^1^ Tanzania adopted Option B+ in 2013, and studies have demonstrated subsequent increases in the rates of HIV diagnosis and linkage to care.^2,3^ However, a meta-analysis of studies in Africa showed that retention in PMTCT care is lower than in the general population of people living with HIV (PLWH), threatening the effectiveness of PMTCT programs and the goals of the Option B+ protocol.^4^ Multiple studies conducted in East Africa have identified HIV stigma as a primary factor influencing retention in PMTCT care.^5–8^

In Tanzania, record reviews of PMTCT programs suggest that 19% of women enrolled in PMTCT did not return after their first visit, and by two years postpartum, 59% of patients were lost to follow up.^2^ Similarly, in a longitudinal study following pregnant WLHIV in the Kilimanjaro region, 21% of participants were identified as having poor care engagement outcomes in PMTCT at six months post-partum.^5^ Qualitative work in Tanzania suggests that HIV stigma is a significant contributor to poor care engagement in PMTCT.^9^

Stigma impacts retention to PMTCT in a variety of ways.^5^ During pregnancy and the postpartum period, WLHIV in Tanzania describe feeling uniquely vulnerable to stigma, particularly in the form of fear of abandonment or mistreatment by their partner or the loss of other key social supports.^6,10,11^ Pregnant WLHIV commonly note manifestations of stigmatizing attitudes in the community, including gossip, social avoidance of the woman and her children, anger and disapproval of family members, and a fear of abuse or abandonment by the father of the child.^8,12–14^ These forms of HIV stigma are unique to pregnant women; therefore, the anticipation of stigma may have a strong impact on pregnant women's willingness to disclose their HIV status, in turn influencing social support for care engagement. PLWH who lack social support systems may miss clinic visits due to a fear of being seen at the clinic or due to an inability to explain a need to go to the clinic.^9^ They may also miss medication doses if they are not able to discreetly take their medication.^9^

Community education and universal access to HIV services have had an impact on community stigma, but stigmatizing attitudes still persist and present a barrier to PMTCT care.^15^ In order to better understand the experience of community stigma among WLHIV in the ante-natal setting, we conducted qualitative interviews with a variety of stakeholders. We aimed not only to understand the dimensions of community-level stigma within this setting, but also to identify opportunities within the ANC setting to mitigate HIV stigma and its unique impact on pregnant and postpartum women. The objectives of this study were three-fold: 1) to explore the drivers and manifestations of HIV stigma, 2) to identify examples of resilience in coping with HIV stigma, and 3) to inform opportunities to address HIV stigma in the ANC setting.

This data can contribute important insights to inform strategies to reduce stigma within the ANC setting and improve HIV prevention and care outcomes among pregnant women, their male partners, and infants.

## METHODS

### Setting

This study was conducted in the Kilimanjaro region of northern Tanzania. In 2016, the HIV prevalence in this region among people aged15-49 was 1.1% among males and 3.1% among females.^16^ In the Kilimanjaro Region, 52 health facilities offer PMTCT services,^17^ following the national guidelines for provision of care.^18,19^

This study was conducted at two government health facilities in the Moshi Urban District of the Kilimanjaro Region. The two study clinics see the largest volume of clients in the region^20^; combined, they serve over 2500 pregnant women per year, with approximately 4.8% living with HIV.^21^ Both facilities have ANC clinics that provide routine HIV testing and counselling for all pregnant women at their first ANC visit. Women are strongly encouraged to bring their male partners to the first visit, so that they can receive HIV testing and counselling together. Pregnant women and partners receive the test result the same day, and those who test positive for HIV receive additional post-test counselling, same-day handoff to the PMTCT clinic (or adult HIV clinic, for partners who test positive), and initiation of ARVmedication. PMTCT services include prescription of maternal ARV prophylaxis or treatment and counselling on infant feeding, as recommended by the Tanzanian Ministry responsible for-Health.^18^ All HIV services are provided free of charge with support from the Tanzanian National AIDS Control Program.

### Sample and Procedures

To understand the experiences of community stigma that may influence PMTCT, we interviewed a variety of stakeholders who were recruited from the ANC setting. Semi-structured in-depth interviews were conducted with 20 pregnant and postpartumWLHIV, 12 pregnant HIV-negative women, and 7 reproductive and child health care (RCH) registered nurses (HIV care nurses and PMTCT coordinators). Two focus group discussions (FGDs) were conducted with 13 community health workers (CHWs), who are clinic-based volunteers who offer education and support for people newly initiating HIV care. WLHIV were recruited from a larger cohort study of pregnant and postpartum WLHIV.^22^ HIV-negative women were recruited from the ANC program at the study clinics, either by phone or in-person when presenting for a routine clinic visit. Healthcare workers and CHWs were recruited in-person at the clinic sites. All participants provided written informed consent prior to their interview. Interviews and FGDs were conducted in Kiswahili by a trained research assistant and duration ranged from 45 to 60 minutes. Interviews were recorded with the participant's consent, and audio files were transcribed and translated into English by research assistants and local translators. All identifying data were removed upon transcription.

### Ethical Consideration

Informed consent was obtained from all individual participants prior to inclusion in the study. All study procedures were conducted in accordance with the ethical standards of the national and institutional research committees. The study received ethical approval from the Tanzanian National Institute for Medical Research (protocol #2183), the Kilimanjaro Christian Medical Centre (protocol #915), and the Duke University Institutional Review Boards (protocol # D0371).

### Interview and Discussion Guides

The interview and discussion guides consisted of a series of open-ended questions reflecting the overarching research objectives, followed by a list of possible probes for further exploration. In the interviews, WLHIV were asked about their past experiences with stigma as well as fears they might have of experiencing stigma in the future. HIV-negative participants were asked to describe their feelings about HIV and how they thought people in their community perceived PLWH. Provider interviews and CHW FGDs explored experiences with providing HIV testing, counselling, and support, the role of stigma in patient decision-making related to uptake of and retention in PMTCT care, and patient experiences of stigma. All participants were also asked about suggestions for opportunities to address HIV stigma more generally, as well as within a hypothetical counselling intervention to be implemented within ANC.

### Analysis

Data were analysed using applied thematic analysis, which is a rigorous and systematic approach to identifying empirically driven themes in qualitative data.^23^ Initially, the first author read a small sample of interviews (n=6) to orient to the data. From this initial reading, four domains of enquiry emerged: drivers of stigmatizing attitudes, manifestations of stigmatizing attitudes, resilience to stigmatizing attitudes, and opportunities to address stigmatizing attitudes. A codebook was created, which defined each domain as a code. Initially, four interview transcripts were coded onto the four domains using QSR International's NVivo software (Version 12).^24^ Each transcript was coded independently by two investigators, and the coded transcripts were compared and discussed to reach consensus on the code definitions and criteria for use. Thereafter, all transcripts were coded in NVivo using the four domain codes.

Additional coding was conducted in two phases. First, inductive themes were identified and applied to the domains using summarizing annotations in NVivo about each coded piece of text. Queries in NVivo retrieved the coded text and the related annotations in each domain. Based on the query output, we identified emerging themes in each domain. Second, child codes were established for emergent themes in each of the domains, and coding was conducted in NVivo to apply the child codes to the text. After the second-round coding, memos were written for each domain and separately for each population (WLHIV, HIV-negative women, and CHWs), in order to organize the emerging themes for comparison. Representative quotes were selected to best capture the data. The memos were reviewed across the participant groups, and themes were synthesized to elucidate commonalities and differences among groups.

## RESULTS

The sample included 20 WLHIV (11 pregnant and 9 post-partum) and 12 HIV-negative pregnant women. Women were on average 29 years old (range: 20-40, with the WLHIV about five years older on average). Nearly all of the HIV-negative women were married, while WLHIV were more commonly unmarried but in a relationship. About half of the women were employed in a salaried position or receiving income through informal activities (e.g., selling vegetables or second-hand clothes). Thirteen (13) CHWs participated in the two FGDs. Additional demographic data can be found in Table 1. A summary of the four domains and emergent subthemes can be found in Table 2.

**TABLE 1:** Demographic Summary of in-Depth Interview Participants

	HIV-negative women (n=12)	Pregnant women living with HIV (n=10)	Postpartum women living with HIV (n=9)
**Age, mean (range)**	26 (20–39)	31 (24–40)	32 (26–39)
**Relationship status**			
Married	11	5	4
Cohabitating/relationship but not	1	4	4
married			
Single	0	0	1
Widow	0	1	0
**Education**			
Form 4 (completion of secondary education)	5	3	2
Some secondary education	0	1	0
Standard 7 (completion of primary education)	5	6	6
Some primary education	0	0	1
Vocational school	1	0	0
**Any employment**	7	4	5

**TABLE 2:** Summary of Emergent Domains and Themes

**Drivers**	Fear of infection through social contact Associations of HIV with physical weakness Associations of HIV with immoral behaviour
**Manifestations**	Gossip Physical and social isolation Changes in intimate partner relationships
**Resilient Individuals**	Healthcare workers Supportive family members and partners Role of education in resilience
**Opportunities**	Education about HIV transmission Normalization of HIV as a chronic illness

### Drivers of stigmatizing attitudes

#### Fear of HIV infection

Fear of acquiring HIV infection through personal contact (e.g., sharing a spoon or a razor for shaving) was described as the primary driver of stigmatizing attitudes. As one participant described: *“They think you have left your infection there…Even the spoon they gave you – after finding that you have (HIV) infection, they will throw away that spoon (WLHIV, postpartum, 31 years old).”* These attitudes often extended to the children of WLHIV. A woman living with HIV recalled: *“People might think, ‘I don't want your child to play with my child.’ [The HIV-positive child] might bite [the HIV-negative child], and then she is infected (WLHIV, postpartum, 29 years old).”* Several participants mentioned that parents tell their children not to play with the children of people who were known or rumored to have HIV, and WLHIV frequently described that they did not want to disclose their HIV status due to concerns that their children would be stigmatized.

At the same time, individuals who associated with a person living with HIV could be labelled as having HIV themselves. A woman living with HIV stated that she was socially isolated by hercommunity simply because she had a friend who others thought had HIV. Even though this was before she herself was diagnosed with HIV, people assumed she had HIV by association:

*“There was this man who was a friend of mine who died, but it was not because he was HIV-infected; after his death, they thought he was infected with HIV, but he was not. So, they assumed that because he was my friend, obviously I would be infected as well (WLHIV, postpartum, 39 years old).”*

#### Perception that HIV is associated with physical weakness

Another common driver of HIV stigma was the perception that HIV infection was associated with dependence on medication and/or physical weakness. Although participants acknowledged that HIV was treatable, some expressed that a fear of being dependent on medications led people to avoid HIV testing or HIV care: *“It's because you will not be able to live without medications. That is what worries them a lot (WLHIV, pregnant, 30 years old).”* This perception that PLWH are physically weak was cited as a driver of “othering”these individuals. HIV was also associated with being unattractive, undesirable, and physically frail, including looking sickly or thin:

*“To them (the community), when they see someone has become thin, they don't even think that maybe they are stressed or depressed, or that the child hasn't gone to school or the child didn't pass the exams. They just say they are done; they have ‘ngoma’ (a pejorative word for HIV) (WLHIV, postpartum, 31 years old).”*

#### Perception that HIV is associated with immoral behaviour

Perceived immorality was also a driver of stigma. One woman living with HIV expressed the sentiment that people who were HIV-negative believed that they were superior to those living with HIV and looked down on those with HIV. She expressed the perception that HIV-negative people believed that PLWH were beneath them, and that they would never engage in such immoral behaviour that could put them at risk of contracting HIV: *“You have already been infected, so they talk about you because they (believe that they) cannot get it (WLHIV, postpartum, 29 years old).”* Additionally, some HIV-negative participants expressed that HIV was a punishment from God for immoral behaviour, and that living with HIV was almost worse than death: *“God has not loved them (PLWH) enough to take them (HIV-negative woman, pregnant, 23 years old).”*

HIV-negative participants often noted that communities associate HIV with sexual promiscuity. This stigmatizing attitude was described as common in faith communities, where people were often judged based on their morality. One HIV-negative woman stated that *“people believe that if one has HIV, they have been maybe prostitutes (HIV-negative woman, pregnant, 30 years old).”* Others expressed fear of being judged if they were diagnosed with HIV, because it could lead others to believe that their partner was unfaithful:

*“When I was told that I was to do an HIV test with my partner, who is [a long-distant truck driver], I said, ‘Oh God, please help me.’ If I have it, it will be an embarrassment, and everyone will talk about it (HIV-negative woman, pregnant, 24 years old).”*

### Manifestations of stigmatizing attitudes

#### Gossip

Gossip was the most commonly noted manifestation of stigmatizing attitudes in the community and was a primary reason that women with HIV did not want to disclose their HIV status to family members or the broader community. Many WLHIV discussed gossip in the form of being pointed at, laughed at, or talked about:

*“They sit in groups and talk about you… they say that you have HIV… I am used to my neighbours – their conversations and types of things they say. I don't mind them anymore. I know that they are my enemies… (WLHIV, postpartum, 31 years old.”*

Another woman recalled community members pointing fingers at her in the context of their belief of her impending death due to HIV: *“They talk a lot, point fingers at you. They say he/she will die tomorrow… (WLHIV, postpartum, 25 years old).”*

In discussing the decision not to disclose one's HIV status, participants frequently spoke about women who chose not to disclose beyond a select few people (e.g., to extended family, neighbours, or workmates) for fear of their status spreading throughout the community via gossip. Several WLHIV identified specific people or groups who would not be able to keep their status a secret:

*“I can't disclose to my workmates because I know when I disclose to one of them in the office, others will know… she will tell another person, and the other person will also tell others. At the end, all of them will know (WLHIV, postpartum, 26 years old).”*

In some instances, there was a perception that people would gossip while drinking alcohol. One woman stated that even if her father were alive, she would not disclose to him because of his drinking: *“I wouldn't have told [my father], because he was an alcoholic and if I disclosed, he could spread it (WLHIV, postpartum, 31 years old).”* Similarly, another woman statedthat she would not disclose to her siblings: *“My young ones and brothers, once you tell them and they are drunk, they can talk about you, or maybe they will tell their wives and their wives tell others (WLHIV, postpartum, 29 years old).”*

The decision to be selective with one's disclosure was often supported by healthcare workers: *“We don't advise you to share the information with everybody, because some people cannot keep secrets. You might share the news with one person and then she turns it into an announcement. You just look for someone whom you can trust with your secrets (Registered RCH Nurse).”*

#### Isolation

Both physical and social isolation were identified as manifestations of stigmatizing attitudes. Physical isolation included using separate dishes or eating separate food from someone with HIV, or not staying at the house of someone with HIV. Social isolation included the loss of friendships and ostracism by family and friends.

Women consistently gave examples of avoidance of physical contact by community members who knew the status of someone living with HIV. One woman living with HIV recalled a change in relationship with an HIV-negative friend, who refused to interact with her any longer after learning of her HIV-positive status: *“We were living very well. When she cooked, she would share with me. But now, even if I cook at my place, I can't give her food, because we would be sharing what we cooked (WLHIV, postpartum, 31 years old).”* One woman living with HIV described a relative who instructed her children to keep their belongings away from hers: *“She said to me that your sister restricts her children from coming to your house because you are sick, and tells them not to put their toothbrush next to yours (WLHIV, postpartum, 39 years old).”* One healthcare worker remarked that patients *“say that they are given special/separate dishes to use exclusively (Registered RCH nurse).”*

WLHIV also cited multiple examples of social isolation through loss of friendships and being ostracized by family and friends. One woman recalled a change in a close friendship: *“Her child was always coming to my room. Then, she told her child not to come interact with me… (now) she doesn't want any relationship with me.…(WLHIV, postpartum, 31 years old).”* Another woman noted that relatives stopped visiting her after she was diagnosed with HIV:

*“They were not telling me directly; they were telling their children. I was staying with my sister's child (15 years old) and they came to take her… They were telling her, ‘don't go to sleep at your aunt's house; she will infect you because we think that she is sick…’ (WILHIV, postpartum, 39 years old)”*

A small number of participants were afraid to disclose to their partners or others because they feared that they would lose care, support, or assistance in the pregnancy or postpartum period. One worker gave an example of a woman living with HIV: *“She's afraid that when she first shares it (her status), she won't get the care she used to… (people will think that) she is already dying (Registered RCH nurse).”*

Healthcare workers recalled two instances of women being kicked out of their houses because of their HIV status. In the case below, a healthcare worker describes how a woman living with her family was evicted.

*“In the end, her family members found out (about her HIV status) … I am not sure if they saw the card or the medicine or what. They sent her away from the house, and she came here and asked for transport fare (to her home village) … There was a time that we were looking for her, but she never came back and we don't know how she is doing (Registered RCH nurse).”*

#### Relationship with Partner

Among healthcare workers, a commonly discussed manifestation of stigma was a change in a patient's relationship with her male partner. Healthcare workers noted that many female patients were fearful of disclosing their status to their male partners for fear of being stigmatized, ostracized, abused, or abandoned. Some shared examples of dissolutions of relationships that occurred once a male partner discovered his partner's HIV status. In some instances, males had tested negative (i.e., the results were discordant); in others, the men refused to get tested themselves. Healthcare workers felt that the anger and abandonment were driven by the perception that the first person to test positive for HIV was the one who *“brought the infection into the relationship,”* and that person was judged to be responsible for the infection.

WLHIV and HIV-negative women gave similar examples of relationship changes that occurred after the women were diagnosed with HIV; they described scenarios including women being abandoned by their partner after receiving an HIV diagnosis, male partners being unfaithful, or couples continuing to live together but the men isolating their partners.

Several healthcare workers noted that when women received the result of their HIV test at the clinic, some were adamant about not disclosing their status to their male partners out of fear that their partners would blame, stigmatize, or leave them:

*“They are afraid that the men will leave them, so they tell you, ‘Sister, if I tell this man, he will leave me, and the man is everything to me… so sister, you see? What do you think will happen if I tell him about it? (Registered RCH nurse)’”*

Nearly all interviewed healthcare workers brought up examples of women who disclosed their HIV status and were then left by their partner. One healthcare worker shared: *“We have discovered that most of the time, discordant results break marriages. Once they get to the gate, they part ways and each of them goes their separate way (Registered RCH nurse).”* Another worker describes the need for careful counselling in diagnosing discordant couples, saying that *“if wisdom is not applied, you could become the first nurse to break a marriage (Registered RCH nurse).”*

Several WLHIV discussed the impact that stigma by male partners had on their wellbeing, leaving them vulnerable during pregnancy and in the postpartum period. A woman recalled the lack of support she received from her partner upon disclosure: *“He left me at the hospital, and he didn't provide any support or pay the hospital bills. It was my mother who came to take me from the hospital (WLHIV, post-partum, 37 years old).”* A healthcare worker recalled a similar abandonment by the man in a discordant couple:

*“She brought him to the clinic, and they tested together. The man tested negative, and the woman was HIV-positive. They received their results well and the man said, ‘there is no problem. I will be taking care of her.’ He was really polite. But before the month was finished, he left her. The woman came to the clinic and said to me, ‘Sister, if I had known this, I wouldn't have brought him to the clinic to be tested.’ She said that she was no longer getting money for support and she didn't have any business that would give her an income. Can you imagine such situations as these? They are really painful (Registered RCH nurse).”*

This healthcare worker also described a woman living with HIV being sent away from her home by her partner as a form of stigma:

*“They arrived home, and the stigma started with the husband. The husband started to stigmatize her. He told her, ‘I don't want to see you in my home anymore. Go to your parents’ house.’ To this day, I haven't seen that lady return to the clinic (Registered RCH nurse).”*

### Resilience to Stigma

Despite the common occurrence of harmful forms of HIV stigma, participants also identified individuals in their community who were resilient to stigmatizing attitudes; that is, individuals who were resistant to and did not perpetuate stigmatizing attitudes. These resilient individuals were typically those who worked in healthcare, family members who knew others living with HIV, and some supportive partners. Examples emerged of how community members developed resilience to stigma from HIV education.

#### Healthcare Workers

Healthcare workers commonly cited their responsibility as service providers as the reason they were role models for resilience to HIV stigma for their patients and community. One healthcare worker stated that she wanted to help patients fight and face stigma because it helped them properly take their medicine. There was a consensus among CHWs in an FGD that it made providers happy to see their clients living well, which drove their efforts to create a supportive and stigma-free environment for clients. Another participant said that her role as a CHW encouraged her to be resilient to HIV stigma in her own home:

*“We do this job in the community, and the community begins in our houses, because this disease has touched the whole community. So, as you give service to the community, you'll find that you already started with your own family (CHW).”*

#### Supportive Family Members and Male Partners

WLHIV cited examples of support they had received from trusted individuals in their lives, most often family members. One participant said that her sister provided emotional support and would help her financially, even though she lived in Kenya: *“When I have a problem, I call her. She asks how I am doing, and if I am taking medication. If I don't have money for milk, I ask her, and she provides (WLHIV, postpartum, 31 years old).”*

Several participants cited their mothers as a source of support. One participant, after being diagnosed with HIV, discovered that her husband had been previously married to someone who had died with HIV, but he had not disclosed his HIV status to her. This participant shared this information with her immediate family, disclosing her status as well. Her family was very protective of her, encouraging her to leave her husband and move in with them to seek HIV treatment:

*“Before I started taking HIV medication, my mother was encouraging. She said, ‘my daughter, don't worry. This has already happened but having the infection does not mean you will die. I will take you to the clinic and you will get counselling and medications. You will be fine, and the baby will be born without infection.’ And she took me to the clinic (WLHIV, postpartum, 25 years old).”*

A few healthcare workers also shared anecdotes of supportive male partners. One healthcare worker shared, *“The husband did not have the infection (HIV), but he said that he was ready to cope.… he said, ‘I vowed to be with her for better or for worse.…I will take care of her until the end.’ (Registered RCH nurse)”*

#### Role of Health Education in Resilience

Among HIV-negative participants, a common theme emerged that knowledge about HIV transmission was a key reason they did not perpetuate stigma in the community. In response to a question about how education helped her learn not to stigmatize others with HIV, one respondent remarked:

*“When I see someone whom others point at because of HIV, I would like to talk to her, so she won't feel lonely. I will just tell her that is a normal situation, and make sure you do this and that… HIV-negative woman, pregnant, 32 years old”*

One CHW gave an example of a husband who isolated his wife when he first learned of her HIV status, but then learned to overcome his own stigmatizing attitudes by learning about HIV: *“(Initially) the husband took another room in the same house, so the man was sleeping in his own room while the wife slept in her own room with the baby. It was as if they were separated (CHW).”* However, after the husband met with the CHW, he came to see that he should not *“judge his wife”* and instead should *“get education on how to live in love even more than before.”*

### Opportunities to Mitigate Stigmatizing Attitudes

Participants were asked to share their ideas about strategies for addressing HIV stigma in ANC. The most common suggestions included normalizing HIV, improving counselling after delivering HIV test results, providing community-wide education on HIV transmission, and sharing examples of community role models who portrayed resilience to HIV stigma.

#### HIV Normalization

Across all interviews, healthcare workers mentioned the value of normalizing HIV, emphasizing that HIV is like other chronic diseases – such as diabetes and hypertension – that require lifestyle modification and daily medication. Healthcare workers believed this concept was important as it would help detach the associations of death and stigma from HIV. One healthcare worker described what she would tell any patient about HIV:

*“I would tell you it's like any other normal illness, and that HIV doesn't kill, it's the opportunistic infections which kill. I will give you an example of the diabetic patients who take pills every day – HIV patients think that they are the only patients who take pills every day, but there are diabetic and hypertensive patients who take their medications every day (Registered RCH nurse).”*

Furthermore, an HIV-negative woman suggested to emphasize that people who test negative for HIV are *“not different from those who are infected.”* More participants stated that interventions to address HIV stigma should speak to human empathy: *“Above all, we should have that feeling of being human, and care for those who are found to be HIV-positive so even if they meet the infected person, they should be able to consider their situation as a normal one (WLHIV, postpartum, 29 years old).”*

A woman living with HIV also stated that it is important for people to recognize that anyone can contract HIV, and people who do not have HIV should recognize their own vulnerability: *“They should just understand it is a normal infection like any other, and it might happen to anyone, even their relatives (WLHIV, postpartum, 37 years old).”*

#### Health Education

WLHIV consistently suggested health education as a method to mitigate HIV stigma, particularly combating misinformed fears relating to the transmission of HIV: *“They should know that HIV transmission occurs through using sharps things, and through unprotected sex, so they should not fear that they will be infected by an HIV-positive person just by sitting with them (WLHIV, postpartum, 25 years old).”* Some HIV-negative women also gave testaments to how receiving health education and information about HIV helped them not stigmatize others.

Healthcare providers recognised the role of counselling to address HIV stigma, particularly in the context of HIV testing; however, interviews documented inconsistency in the content of counselling during HIV testing in the study clinics. Some clients received information on how to prevent future transmission of HIV, but most HIV-negative women responded that they did not receive any counselling or information after they received their test result. One CHW suggested that education should be provided regardless of whether someone tests positive or negative, *“so that everybody will understand well what causes the infection.”* This CHW also emphasized that efforts were needed to reach those who cannot access education via media or newspaper:

*“If we were empowered and able to reach all the people and be giving out the education… at least what we know, it would help. But how do we reach all these people to tell them what they need to know? That is a little difficult (CHW).”*

A CHW suggested that CHWs could bring education to households, going door-to-door, disseminating information about how HIV is transmitted, encouraging people to get tested for HIV, and explaining that people should not stigmatize others living with HIV. The CHWs said that one household can often have 6-7 rooms and many people, making it possible to share information with large numbers of people in just one visit.

Finally, across all populations, participants suggested that as part of HIV education, people should be presented with examples of behaviours of others who do not stigmatize people with HIV. For example, one HIV-negative women suggested that people *“should see a picture of someone eating food with another one who is infected (HIV-negative woman, pregnant, 28 years old).”*

## DISCUSSION

Stigma has a clear impact on HIV care and engagement and takes an emotional toll on PLWH.^25,26^ The various forms of stigma experienced by PLWH emanate from community-level stigmatizing attitudes, making these attitudes important to study. In this study, perceived drivers of stigma most commonly included a fear of HIV infection through casual contact, and damaging associations of HIV with sexual promiscuity, death, and poor personal choices or moral weakness. These drivers suggest that stigma is not only connected to judgments of the disease as dangerous, but also negative judgments about the behaviours of PLWH. Manifestations of stigma most commonly included gossip, social isolation, and changes in relationships with male partners. These forms of enacted stigma clearly link to barriers to care engagement for PLWH, particularly for pregnant women who need both HIV care and attentive antenatal and postpartum care.

Our study found that stigma was cited as a strong reason that WLHIV felt they could not disclose their HIV status to family members or male partners. It is well documented that non-disclosure impacts care engagement: PLWH who have not disclosed their status to someone are at risk of missing clinic visits (if they are unable to explain the visit to family or an employer) and missing medication doses (if they are unable to discreetly take pills and get to the clinic for refills).^14,27^ However, pregnant WLHIV face additional barriers to care. Because of inequities in socioeconomic power in relationships with male partners, women may struggle to find transportation fare to reach a clinic, even if they have an independent source of income. For pregnant women with other children at home, they may experience challenges in securing childcare while they go to the clinic, and those who have not disclosed their status may struggle to explain their absence from the home.^27–29^

Many participants noted that male partners often perpetrated stigma against HIV-positive female partners, mainly by withholding financial support. This may be particularly true in contexts where gender inequity is high and traditional gender roles of the “male as provider” can be detrimental to women's wellbeing.^30^ As discussed by healthcare workers, women whose partners exile them are often lost to care, as they move to a different place to live with family, and often are not able to continue receiving care at their original clinic, if at all. Providers should therefore be more attuned to the vulnerabilities of pregnant WLHIV and help these women plan for transfer of care, in case an interruption may happen due to enacted stigma.

At the same time, examples of resilience to stigma emerged in the form of some supportive male partners and family members, which confirms that stigma from partners is not inevitable. Looking to future interventions, reducing stigma among male partners is crucial to building support for care engagement and emotional wellbeing.^9,21,26^ There is a critical need to develop efficacious interventions to address HIV stigmatizing attitudes in the general population, in order to create a supportive social environment for people seeking HIV care.^5^ A systematic review and meta-analysis of the effectiveness of HIV stigma reduction interventions found that most interventions (which were education-based or included peer-led approaches) led to only small improvements in HIV-related knowledge and small reductions in negative attitudes towards PLWH.^15^ None of the studies were specific to the impact of stigma or anticipation of stigma during the pregnancy period. Furthermore, because the manifestations of stigma we found are consistent with previous documentations of stigma, interventions addressing HIV stigmatizing attitudes should go beyond educational content. Interventions can utilize stigma-resilient individuals as peer facilitators and role models, particularly to encourage male partners to test for HIV and support their pregnant partners to initiate ANC and if they test positive, to initiate PMTCT.

Given universal HIV testing during first ANC, there is an opportunity to address HIV stigmatizing attitudes in the ANC infrastructure. Participants reported inconsistencies in HIV pre-test and post-test counselling and health education, with some saying that they received minimal pre-test information on HIV prevention or the implications of a positive test. Similar inconsistences have been noted in post-test counselling among those who test positive, with missed opportunities for education about pregnancy and HIV.^31,32^ A discussion of HIV stigma and HIV normal-isation could be incorporated into pre-test and post-test counselling to address both personal and community-level stigma. This gap in ANC standard of care represents an opportune place to disseminate information relating to HIV transmission and HIV stigma. ANC also provides an opportunity to provide counselling to serodiscordant couples, in order to help them understand and accept each other's HIV status and to develop strategies to support one another.

To address drivers of stigmatising attitudes, education that dispels myths about HIV transmission should continue to be improved upon and disseminated. Individuals in our sample suggested that HIV stigma could be addressed through health education and added that such education should normalize HIV as a treatable chronic illness like diabetes or hypertension. This educational programming could also include information about HIV transmission that rectifies myths about promiscuity and poor lifestyle choices, as a large driver of stigmatizing attitudes was the association of HIV with moral weakness.

These recommendations were mentioned consistently across all participant groups, suggesting that this messaging could be effective in addressing stigmatising attitudes in the community and also help relieve internalized stigma for PLWH. Educational campaigns and public messaging around HIV that go beyond providing information about HIV transmission are needed to address the drivers of stigma related to the damaging associations of HIV with sexual promiscuity, immorality, physical weakness, and death. Interventions and public messaging that foreground the humanity of PLWH and normalise HIV can help people build empathy and advocacy for PLWH, in turn creating supportive environments for PLWH in the community and in healthcare settings.^21^

Some participants suggested that people might benefit from seeing examples of stigma-resilient individuals in the community, such as an HIV-negative person eating with a person living with HIV. Such role models could help reduce community stigmatising attitudes through peer groups at ANC or through public campaigns and could help show that PLWH are not less human than HIV-negative people. Emergent themes of resiliency, in the form of supportive male partners and family members, showed that the most impactful way to address someone's stigmatizing attitudes could be by addressing attitudes in their innermost circle. This would include incorporating HIV stigma reduction information into couples’ HIV testing and counselling, addressing the prevalent changes in relationships that women documented throughout this study, and helping seronegative partners build empathy for seropositive partners. Further, group counselling that includes both PLWH and HIV-negative individuals could be effective in challenging stigmatising attitudes and potentially reducing stigma while improving social support and care engagement for PLWH.^29,33,34^

### Limitations

Although this study used a comprehensive interview guide and interviewed a variety of stakeholders in ANC (WLHIV, HIV-negative women, and healthcare workers) to capture nuances of HIV stigma at the community level, the following limitations should be considered. We did not interview male partners, so we were not able to capture men's perspectives on HIV stigma, which might have proven useful for understanding HIV stigma, especially at the level of the family unit. Participants may have altered their responses due to social desirability bias, such as hiding their own stigmatizing attitudes related to PLWH. Data were collected at two clinic sites in a single region of Tanzania and should be generalized with caution.

## CONCLUSION

HIV stigmatizing attitudes create barriers to a woman's ability to fully engage in PMTCT care. Manifestations of stigma such as gossip and loss of support from family and partners can leave pregnant WLHIV particularly vulnerable to challenges in care engagement. Ongoing work to increase community-wide HIV education that normalizes HIV as a chronic disease and challenges drivers of HIV stigma rooted in myths regarding HIV transmission and immorality should continue in order to reduce community-wide stigmatizing attitudes. HIV stigma should continue to be prioritized in a comprehensive response to HIV prevention and treatment, and the ANC environment, which is a site of routine HIV testing, should be seen for its potential to address HIV stigma. Pre-test counselling in ANC clinics should include specific content that addresses HIV stigma, taking advantage of the heightened emotions of an HIV test to build empathy for PLWH. Post-test counselling should reinforce stigma reduction messages for individuals who test negative and should explicitly address both internalized and anticipated stigma for individuals who test positive for HIV, especially those in discordant relationships. Providers should be keenly attuned to the social support resources that pregnant WLHIV have at their disposal. Providers should be trained to identify women with low social support networks and make referrals to other care facilities to prevent disruption of PMTCT care as needed. In addition to such policy, interventions and infrastructure should be built to incorporate peer-led stigma-specific programming into ANC, as examples of healthcare workers, family, and intimate partners who demonstrate and propagate resilience to stigmatizing attitudes in the community exist and could serve as role models to reduce stigmatizing attitudes.

## References

[1] Consolidated Guidelines on the Use of Antiretroviral Drugs for Treating and Preventing HIV Infection: Recommendations for a Public Health Approach. 2nd ed. World Health Organization; 2016. Accessed October 4, 2019. http://www.ncbi.nlm.nih.gov/books/NBK374294/27466667

[2] Cichowitz C, Mazuguni F, Minja L, et al. Vulnerable at Each Step in the PMTCT Care Cascade: High Loss to Follow Up During Pregnancy and the Postpartum Period in Tanzania. AIDS Behav. 2019;23(7):1824-1832. doi:10.1007/s10461-018-2298-8. Published online October 16, 2018. Medline 30327997 PMC6467690

[3] Mazuguni F, Mwaikugile B, Cichowitz C, et al. Unpacking Loss to Follow-Up Among HIV-Infected Women Initiated on Option B+ In Northern Tanzania: A Retrospective Chart Review. 1. 2019;3(1):6-15. 10.24248/EAHRJ-D-18-00025 PMC8279164 34308190

[4] Knettel BA, Cichowitz C, Ngocho JS, et al. Retention in HIV Care During Pregnancy and the Postpartum Period in the Option B+ Era: A Systematic Review and Meta-Analysis of Studies in Africa. J Acquir Immune DeficSyndr. 2018;77(5):427-438. doi:10.1097/QAI.0000000000001616. Medline PMC5844830 29287029

[5] Watt MH, Cichowitz C, Kisigo G, et al. Predictors of postpartum HIV care engagement for women enrolled in prevention of mother-to-child transmission (PMTCT) programs in Tanzania. AIDS Care. 2019;31(6):687-698. doi:10.1080/09540121.2018.1550248. Medline 30466304 PMC6443456

[6] Buregyeya E, Naigino R, Mukose A, et al. Facilitators and barriers to uptake and adherence to lifelong antiretroviral therapy among HIV infected pregnant women in Uganda: a qualitative study. BMC Pregnancy Childbirth. 2017;17(1):94. doi:10.1186/s12884-017-1276-x. Medline 28320347 PMC5360052

[7] Gesesew HA, Tesfay Gebremedhin A, Demissie TD, Kerie MW, Sudhakar M, Mwanri L. Significant association between perceived HIV related stigma and late presentation for HIV/AIDS care in low and middle-income countries: A systematic review and meta-analysis. PLoS One. 2017;12(3):e0173928. doi:10.1371/journal.pone.0173928. Medline 28358828 PMC5373570

[8] McMahon SA, Kennedy CE, Winch PJ, Kombe M, Killewo J, Kilewo C. Stigma, Facility Constraints, and Personal Disbelief: Why Women Disengage from HIV Care During and After Pregnancy in Morogoro Region, Tanzania. AIDS Behav. 2017;21(1):317-329. doi:10.1007/s10461-016-1505-8. Medline 27535755 PMC5218979

[9] Knettel BA, Minja L, Chumba L, et al. Serostatus disclosure among a cohort of HIV-infected pregnant women enrolled in HIV care in Moshi, Tanzania: A mixed-methods study. SSM - Population Health. Published online 2019:100323. doi:10.1016/j.ssmph.2018.11.007 PMC6289955 30560196

[10] Earnshaw VA, Smith LR, Chaudoir SR, Amico KR, Copenhave MM. HIV stigma mechanisms and well-being among PLWH: a test of the HIV stigma framework. AIDS Behav. 2013;17(5):1785-1795. doi:10.1007/s10461-013-0437-9. Medline 23456594 PMC3664141

[11] Sariah A, Rugemalila J, Protas J, et al. Why did I stop? And why did I restart? Perspectives of women lost to follow-up in option B+ HIV care in Dar es Salaam, Tanzania. BMC Public Health. 2019;19(1):1172. doi:10.1186/s12889-019-7518-2. Medline 31455306 PMC6712622

[12] Ezebuka O, Sam-Agudu N, Erekaha S, Dairo M. Correlates of intimate partner violence among HIV-positive women in south west Nigeria. Lancet Glob Health. 2015;3:S23. doi:10.1016/S2214-109X(15)70142-7.

[13] Geubbels E, Williams A, Ramaiya A, Tancredi D, Young S, Chantry C. HIV status disclosure among postpartum women in rural Tanzania: predictors, experiences and uptake of a nurse-facilitated disclosure intervention. AIDS Care. Published online January 24, 2018:1-9. doi:10.1080/09540121.2018.1428724 PMC6250839 29363340

[14] McGrath CJ, Singa B, Langat A, et al. Non-disclosure to male partners and incomplete PMTCT regimens associated with higher risk of mother-to-child HIV transmission: a national survey in Kenya. AIDS Care. Published online November 11, 2017:1-9. doi: 10.1080/09540121.2017.1400642 PMC5895526 29130333

[15] Mak W WS, Mo P KH, Ma G YK, Lam MYY. Meta-analysis and systematic review of studies on the effectiveness of HIV stigma reduction programs. Soc Sci Med. 2017;188:30-40. doi:10.1016/j.socscimed.2017.06.045. Medline 28704645

[16] Tanzania Commission for AIDS, Zanzibar AIDS Commission. Tanzania HIV Impact Survey (A Population-Based HIV Impact Assessment) 2016–2017.; 2018:404.

[17] Mkenda TB, Nokoe KS, Karoki S. Statistical Update on the Implementation of HIV/AIDS Guidelines and Policies in Kilimanjaro Region-Tanzania. Health Sci J. 2019;13(2). https://www.hsj.gr/abstract/statistical-update-on-the-implementation-of-hivaids-guidelines-and-policies-in-kilimanjaro-regiontanzania-24265.html.

[18] Tanzania Ministry of Health and Social Welfare. National Guidelines for Comprehensive Care Services for Prevention of Mother-to-Child Transmission of HIV and Keeping Mothers Alive.; 2013.

[19] Tanzania National Guidelines for the Management of HIV and AIDS. The United Republic of Tanzania Ministry of Health and Public Wellfare; 2015. https://aidsfree.usaid.gov/sites/default/files/04_11_2016.tanzania_national_guideline_for_management_hiv_and_aids_may_2015._tagged.pdf

[20] Msuya SE, Uriyo J, Hussein T, et al. Prevention of mother-to-child HIV transmission at primary health care level in Moshi urban Tanzania: uptake challenges and transmission rate. Global Journal of Medicine and Public Health. 2014;3(1).

[21] Watt MH, Knettel BA, Knippler ET, et al. The development of Maisha, a video-assisted counseling intervention to address HIV stigma at entry into antenatal care in Tanzania. Eval Program Plann. 2020;83:101859. doi:10.1016/j.evalprog-plan.2020.101859. Medline 32795711 PMC7686260

[22] Watt MH, Knippler ET, Minja L, et al. A counseling intervention to address HIV stigma at entry into antenatal care in Tanzania (Maisha): study protocol for a pilot randomized controlled trial. Trials. 2019;20(1):807. doi:10.1186/s13063-019-3933-z. Medline 31888700 PMC6937735

[23] Guest G, MacQueen KM, Namey EE. Applied Thematic Analysis. Sage. 2012.DOI:10.4135/9781483384436

[24] NVivo: Qualitative Data Analysis. QSR International Pty Ltd.; 2018.

[25] Catz SL, Kelly JA, Bogart LM, Benotsch EG, McAuliffe TL. Patterns, correlates, and barriers to medication adherence among persons prescribed new treatments for HIV disease. Health Psychol. 2000;19(2):124-133. doi:10.1037/0278-6133.19.2.124. Medline 10762096

[26] Watt MH, Knippler ET, Knettel BA, et al. HIV Disclosure Among Pregnant Women Initiating ART in Cape Town, South Africa: Qualitative Perspectives During the Pregnancy and Postpartum Periods. AIDS Behav. 2018;22(12):3945-3956. doi:10.1007/s10461-018-2272-5. Published online September 8, 2018. Medline 30196332 PMC6209531

[27] Chinkonde JR, Sundby J, Martinson F. The prevention of mother-to-child HIV transmission programme in Lilongwe, Malawi: why do so many women drop out. Reprod Health Matters. 2009;17(33):143-151. doi:10.1016/S0968-8080(09)33440-0. Medline 19523591

[28] Ekama SO, Herbertson EC, Addeh EJ, et al. Pattern and determinants of antiretroviral drug adherence among Nigerian pregnant women. J Pregnancy. 2012;2012:1-6. doi:10.1155/2012/851810. Medline PMC3317105 22523689

[29] Tam M, Amzel A, Phelps BR. Disclosure of HIV serostatus among pregnant and postpartum women in sub-Saharan Africa: a systematic review. AIDS Care. 2015;27(4):436-450. doi:10.1080/09540121.2014.997662. Medline 25636060

[30] Suandi D, Williams P, Bhattacharya S. Does involving male partners in antenatal care improve healthcare utilisation? Systematic review and meta-analysis of the published literature from lowand middle-income countries. Int Health. 2020;12(5):484-498. doi:10.1093/inthealth/ihz073. Medline 31613327 PMC11701106

[31] Gugsa S, Potter K, Tweya H, et al. Exploring factors associated with ART adherence and retention in care under Option B+ strategy in Malawi: A qualitative study. PLoS One. 2017;12(6):e0179838. doi:10.1371/journal.pone.0179838. Medline 28636669 PMC5479573

[32] Katirayi L, Namadingo H, Phiri M, et al. HIV-positive pregnant and postpartum women's perspectives about Option B+ in Malawi: a qualitative study. J Int AIDS Soc. 2016;19(1):20919. doi:10.7448/IAS.19.1.20919. Medline 27312984 PMC4911420

[33] Ma Q, Tso LS, Rich ZC, et al. Barriers and facilitators of interventions for improving antiretroviral therapy adherence: a systematic review of global qualitative evidence. J Int AIDS Soc. 2016;19(1):21166. doi:10.7448/IAS.19.1.21166. Medline 27756450 PMC5069281

[34] Watt MH, Aronin EH, Maman S, Thielman N, Laiser J, John M. Acceptability of a group intervention for initiates of antiretroviral therapy in Tanzania. Glob Public Health. 2011;6(4):433-446. doi:10.1080/17441692.2010.494162. Medline 20635269 PMC3530384

